# Osteosarcoma of the mandible: A case with a clinical appearance similar to benign lesions

**DOI:** 10.1002/ccr3.7198

**Published:** 2023-04-12

**Authors:** Atessa Pakfetrat, Toktam Zamani, Najmeh Anbiaee, Majid Mirhashemi, Elahe Vazavandi

**Affiliations:** ^1^ Oral and Maxillofacial Diseases Research Center Mashhad University of Medical Sciences Mashhad Iran; ^2^ Professor of oral and maxillofacial medicine, Oral and Maxillofacial Diseases Research Center Mashhad University of Medical Sciences Mashhad Iran; ^3^ Assistant of oral and maxillofacial Medicine Mashhad University of Medical Sciences Mashhad Iran; ^4^ Assistant professor of oral and maxillofacial radiology, Oral & Maxillofacial radiology Department Mashhad University of Medical Sciences Mashhad Iran; ^5^ Associate professor of oral and maxillofacial pathology, Oral & Maxillofacial pathology Department Mashhad University of Medical Sciences Mashhad Iran; ^6^ Associate professor of oral and maxillofacial Medicine, Oral & Maxillofacial medicine Department Kerman University of Medical Sciences Kerman Iran

**Keywords:** benign lesions, diagnosis, mandible, osteosarcoma

## Abstract

A 34‐year‐old woman with complaints of mandibular swelling that started 4 months earlier was referred to the Oral Diseases Department. Based on the clinical and radiographic appearance, the primary diagnosis was an intraosseous reactive lesion. However, the result of histopathology indicated osteosarcoma.

## Abstract 2

The diagnosis of osteosarcoma is sometimes challenging due to similarity of its clinical manifestation to those of benign lesions. Correct diagnosis and timely referral have a major impact on prognosis and patient survival.

## 1 INTRODUCTION

Osteosarcoma is the most common primary malignant bone tumor occurring mainly in long bones such as the femur and tibia.^1^ Osteosarcoma is divided into three categories, namely central, superficial and extraosseous (rare). It occurs primarily in adolescence and after the age of 60, and is almost more common in women. Intraosseous malignancy is the second most common malignancy in the jaw and occurs mainly in the mandible.^2^ The etiology of osteosarcoma is not yet clear. The most common clinical symptoms of osteosarcoma are swelling in the jaw and pain in the long bones. This neoplasm usually has a diverse radiographic and histopathologic appearance. The radiographic appearance of osteosarcoma can be radiolucent, radiopaque, or both. Sunburst pattern and Codman triangle are the classic appearance of this sarcoma, although it is not pathognomonic. On the other hand, PDL widening can be one of the early signs of osteosarcoma. The distinctive feature of the present case report is the diagnosis of early‐stage osteosarcoma, which can have an appearance similar to that of benign‐looking lesions.^3^ Other cases of the clinical resemblance of osteosarcoma to an early‐stage reactive benign lesion have also been reported.^4^

Here, we report the case of a 34‐year‐old woman whose clinical and radiographic appearance of the lesion closely resembled that of a central giant cell granuloma (CGCG), which is a reactive lesion, with the histopathologic appearance of an osteosarcoma.

## 2 CASE REPORT

A 34‐year‐old woman with complaints of gingival swelling and loosening of the lower right canine was referred to the Oral Diseases Department, Faculty of Dentistry, Mashhad University of Medical Sciences, Iran, in July 2021. The patient had no particular habits, no systemic disease or taking certain medications and his psychological status was normal. 4 months earlier, the patient was referred to a general dentist with pain in the lower right area and had a pulpotomy of the lower right fourth tooth. The patient also noted a brief loosening of the same tooth 20 days later and an exophytic lesion in the vestibule of the area. On the second referral to a general dentist and with a possible diagnosis of dental infection, the patient was treated with the antibiotics cephalexin and metronidazole for 2 weeks with no improvement. According to the patient, the size of the lesion almost tripled in 4 months. On extraoral examination by an oral and maxillofacial specialist, facial asymmetry was observed in the form of a brief swelling with a firm consistency in the left cheek toward the lower border of the mandible. No lymph node involvement was observed on examination of the neck.

**FIGURE 1:**
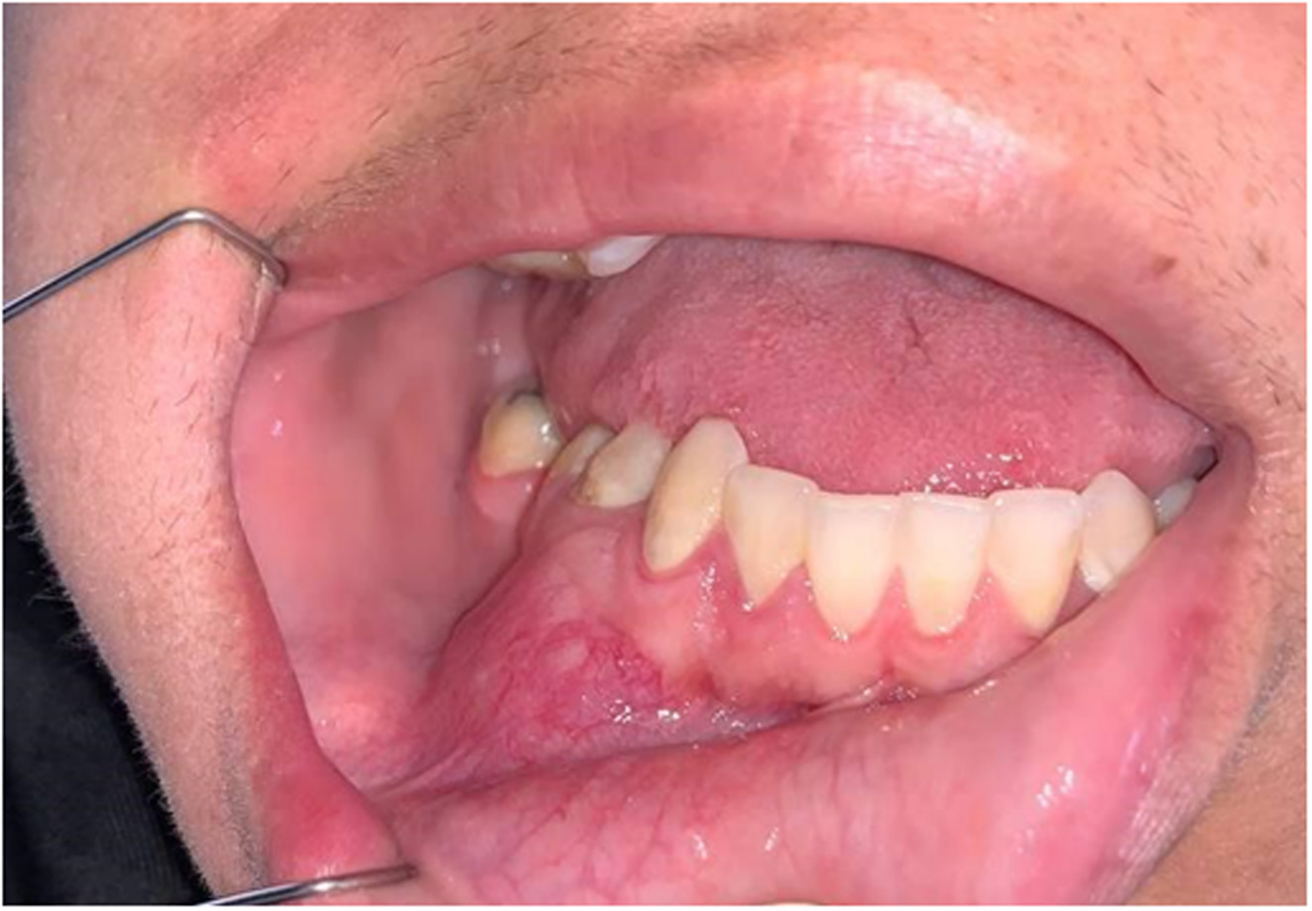
Clinical appearance: a dome‐shaped exophytic lesion with intact mucosa in the buccal vestibule of the right mandible.

On intraoral examination, there was an exophytic dome‐shaped lesion with an approximate size of 2.5 × 3 cm in the buccal vestibule from the distal lower right second tooth to the distal lower right fifth tooth. The color of the mucosa covering the lesion was normal with some telangiectasia. The lesion was uniformly firm with bone thinning noticeable upon touch in some areas. The lower right third tooth showed a grade 2 loosening and the lower right fourth and fifth teeth showed a grade 1 loosening. The patient also had pain upon touch and dental occlusion. In the lingual area, a short extension from the distal lower right second tooth to the mesial lower right sixth tooth with a hard consistency was observed (Figure 1).

After taking the medical history and clinical examination, a clinical diagnosis of reactive lesions such as aggressive CGCG due to the higher prevalence and next mesenchymal tumors were considered. Consequently, panoramic X‐ray and CBCT of the posterior mandible were prescribed.

**FIGURE 2:**
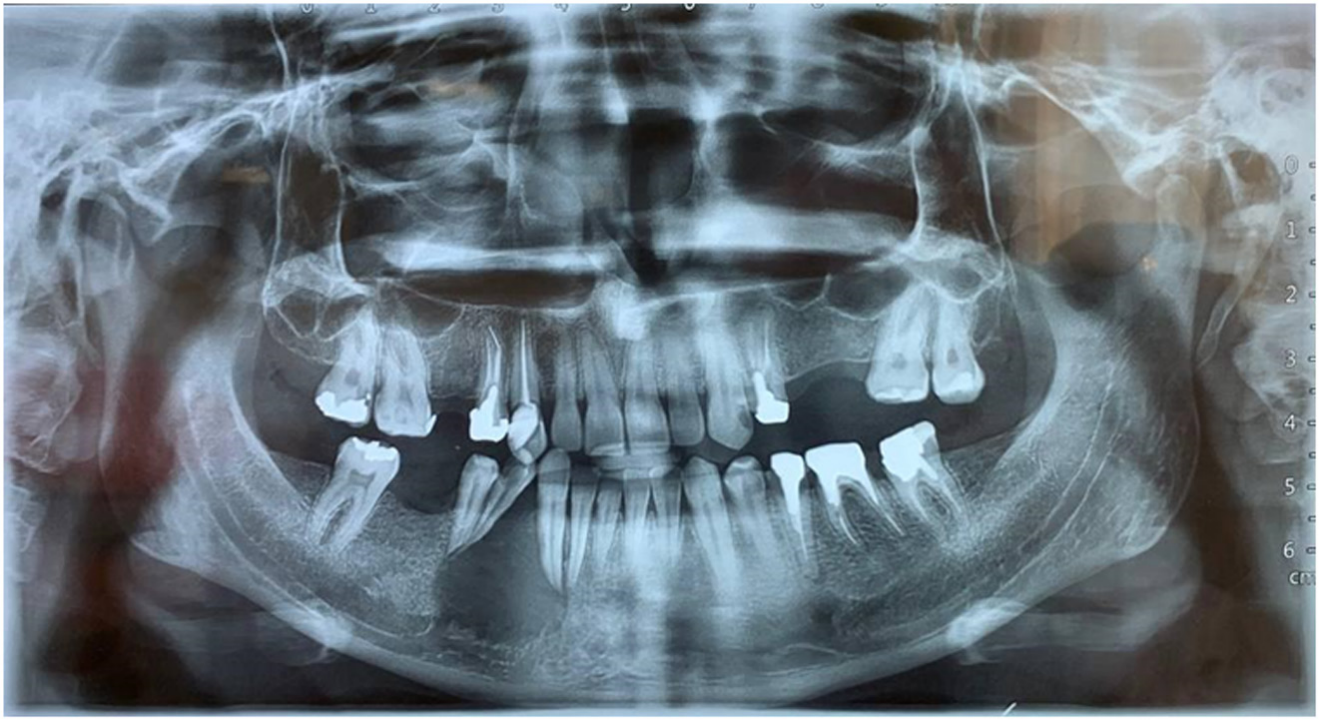
Panoramic appearance: poorly defined radiolucent lesion in the lower right third and fifth teeth, fine bone structure inside the lesion and band‐like PDL widening in the distal lower right second tooth and the mesial lower right third tooth are visible. In addition, the displacement of the lower right third to fifth teeth, their root erosion and the destruction of the upper border of the lower right alveolar canal can be viewed.

**FIGURE 3:**
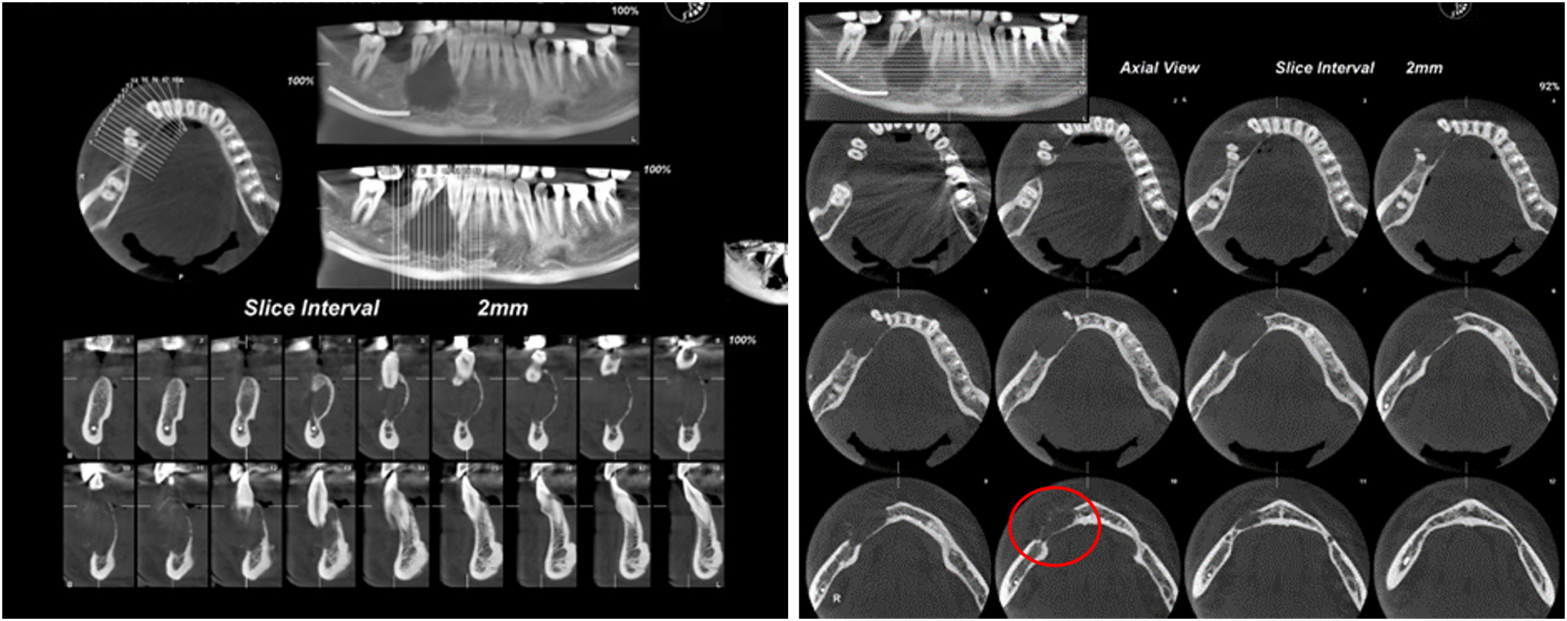
CBCT appearance: a radiolucent lesion with distinct borders, multilocular wispy (cut no. 10) along with perforation of the buccal and lingual cortex, root resorption and tooth displacement are visible.

On the panoramic appearance, an ill‐defined radiolucent lesion was observed in the lower right third and fifth teeth, where the fine bone within the lesion was visible. In addition, there was a band‐like PDL widening in the distal lower right second tooth and the mesial lower right third tooth along with the displacement of the lower right third to fifth teeth and their root erosion. The upper border of the lower right alveolar canal was also visible. On the CBCT appearance of the radiolucent lesion with distinct borders, multilocular wispy (Figure 3, cut no. 10) was observed along with buccal and lingual cortex perforation, root resorption and tooth displacement (Figures 2 and 3).

Aggressive CGCG, juvenile COF (radiolucent phase) and osteosarcoma were respectively considered in the differential diagnosis.

**FIGURE 4:**
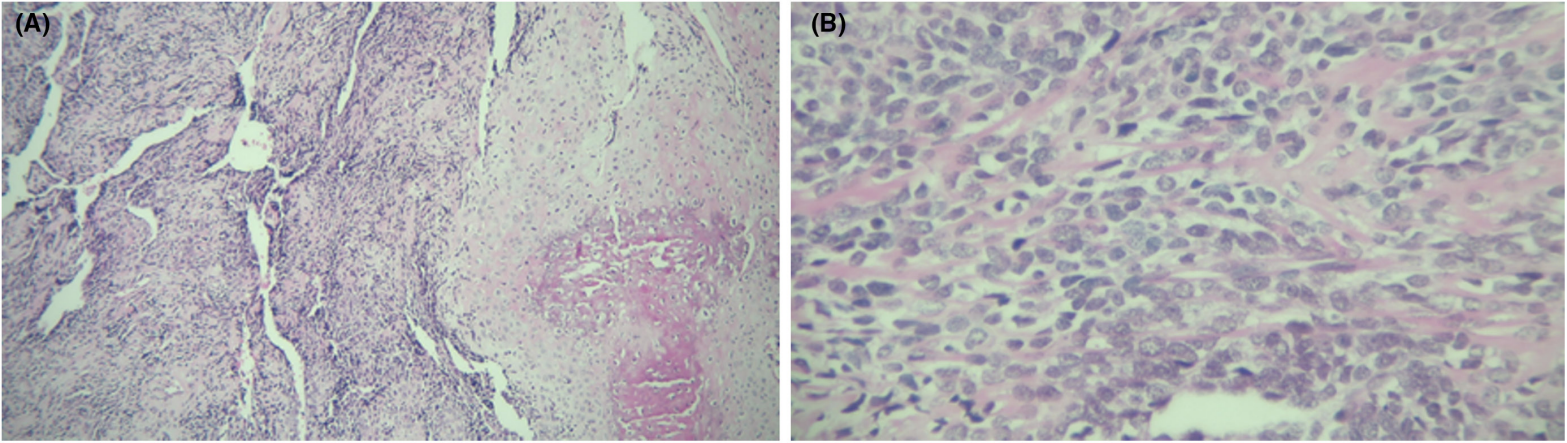
Histopathological appearance (H&E staining): an image with 20× magnification showing malignant hyperchromatic cells and chondroid areas (A). An image with 40× magnification showing the osteoid areas within the malignant cells (B).

The patient was referred to a maxillofacial surgeon for an incisional biopsy. The biopsy from the right mandibular buccal vestibule was sent to the laboratory in the form of two soft tissue pieces with an elastic consistency and a total size of 20 × 5 × 15 mm. Malignant proliferation of mesenchymal cells with osteoid production was observed microscopically by an oral and maxillofacial pathologist. The cells had pleomorphism, hyperchromatism, mitosis, and bizarre nuclear and cytoplasmic shapes. Osteoid and, in some areas, chondroid matrix were observed within the cells. Eventually, a chondroblastic osteosarcoma lesion was diagnosed histopathologically (Figure 4).

The decision was therefore made in favor of completely removing the lesion by an oral surgeon followed by chemotherapy. Upon removal of the lesion, a large 2 × 4 × 6 cm piece was sent to the lab along with the lower right third to fifth teeth. In the microscopic appearance, episodes of neoplastic sarcoma consisting of spindle to round cell proliferation with moderate to severe cytonuclear atypia and atypical mitosis with osteoid and chondroid formation were observed by the general pathologist. Again, it was diagnosed histopathologically as a high‐grade sarcoma consistent with osteosarcoma.

After confirming the diagnosis of osteosarcoma in completely removing the lesion, the patient was referred to an oncologist. Lung CT scan, bone nuclear scan and orbital scan were performed. A 6 mm diameter lytic lesion in the superior lateral bone of the left orbital bone was observed on orbital scan by an interventional radiologist who diagnosed the lesion in favor of metastasis. On the recommendation of the oncologist, 12 chemotherapy sessions were prescribed and the patient was then referred to an oral surgeon for mandibular resection. The patient successfully completed the chemotherapy sessions and did not require mandibular resection after assessment by PET scan and only underwent a 3‐month follow‐up.

## 3 DISCUSSION AND CONCLUSION

Osteosarcoma is a highly malignant tumor with a high potential for destruction. It often occurs in the second decade of life, although it also occurs after the age of 50. Studies report different mean ages for onset of osteosarcoma. About 80% of osteosarcoma cases are of internal bone origin and only 6%–8% occur in the orofacial bones, making it a relatively rare malignancy. Nevertheless, the most common sarcoma occurs in the jaw followed by chondrosarcoma.^5^ In Krishnamurthy's 2017 cohort study of 17 patients with osteosarcoma of the jaw, the mean patient age was 37 years and a higher prevalence in females as well as a higher proportion of mandibular than maxillary involvement were reported.^6^ In Ogunlewe's study over 21 years, the mean age for osteosarcoma of the jaw was 27.2 years.^7^ In general, osteosarcoma is slightly more common in males than females (1.3:1) and affects the mandible and maxilla almost equally. In the mandible, however, it is more common in the ventral, parasitoid, symphysis, and ramus regions.^8^ Tanzawa studied 114 cases with osteosarcoma of the jaw, of which 68 cases had symptoms of swelling, whereas swelling was accompanied by pain in only three cases.^9^ In our patient, both swelling and pain were the primary symptoms.

Osteosarcoma has a number of radiographic appearances, including radiopaque, completely radiolucent with ill‐defined border, and mixed. The classical periosteal reaction sunburst appearance can also be observed in osteosarcoma of the jaw, and in some cases, Codman triangle (triangular elevation of the periosteum), spiking root resorption, Garrington sign, and PDL widening are also noticeable.^4^

In the study by Fernandes et al., among 11 cases of osteosarcoma of the jaw, five cases had PDL widening and six cases had a sunburst appearance.^10^ Elkordi et al. studied 21 patients with osteosarcoma of the jaw and indicated that only two cases had sunburst appearance.^8^ Lidquist et al. also reported the sunburst appearance along with PDL widening as the pathognomonic appearance of osteosarcoma of the jaw.^4^

We did not notice a clear sunburst appearance in our patient (Figure 3). In fact, the diagnosis was challenging both clinically and radiographically. In the clinical appearance, we noticed a localized swelling and a course lasting several months, which mostly indicated reactive lesions. In the panoramic appearance, tooth root analysis and root divergence suggested benignity and band‐like PDL widening of the distal second tooth and the mesial third tooth together with the ill‐defined borders of the lesion suggested malignancy. In the CBCT images, the well‐defined borders of the lesion and the absence of a clear periosteal reaction were in favor of benignity.^11^

Because some reactive lesions are aggressive, they can be differentially diagnosed as malignant lesions. One such fast‐growing reactive lesion is aggressive CGCG, which in our case was considered as the first differential diagnosis. According to the study by Najar Karimie et al. in 2020, aggressive CGCG lesions mostly occur in women under 30 years of age and in the anterior mandible. Moreover, due to the high growth rate, severe swelling, pain, tooth loosening, root resorption, and cortex perforation, they can be mistaken for a malignant lesion. All such characteristics were similar to our case.^12^

In their 2002 review, Stave et al. evaluated 232 cases of CGCG, most of which had an unclear and multilocular border. Additionally and consistent with our case, most cases had bone pain, expansion and tooth displacement.^13^

Jeiraj et al. in 2019 addressed some characteristics of aggressive CGCG in distinguishing it from bone malignancies. Age below 30 years, tendency to the front of the mandible, visible bluish lesion behind the mucosa, and blood aspiration in FNA can help distinguish CGCG from malignant lesions such as osteosarcoma. In our case, in addition to the location of the lesion, which was in front of the mandibular molars and similar to CGCG, the absence of three other characteristics could facilitate the definitive diagnosis.^14^

On the other hand, early‐stage osteosarcoma can be mistaken for other benign lesions. In their case report, Arnold et al. concluded in 2020 that osteosarcoma can be easily confused with benign fibrous lesions such as fibrosing dysplasia, cemento‐osseous dysplasia and ossifying fibroma, especially in cases of low‐grade osteosarcoma where osteoid is barely visible on histopathological appearance.^15^

Because of the potential clinical and radiographic similarities between osteosarcoma and reactive lesions, clinicians should exercise caution in diagnosis and adopt an appropriate and timely approach. The decisions should be made as a team including Oral and maxillofacial medicine specialists, Oral and maxillofacial radiologists and pathologists, Oral and maxillofacial surgeons.

## AUTHOR CONTRIBUTIONS

**Atessa Pakfetrat:** Conceptualization; project administration; supervision; visualization. **Toktam Zamani:** Conceptualization; project administration; writing – original draft; writing – review and editing. **Najmeh Anbiaee:** Data curation; methodology; supervision. **Majid Mirhashemi:** Methodology; supervision. **Elahe Vazavandi:** Data curation; writing – original draft.

## FUNDING INFORMATION

None.

## CONFLICT OF INTEREST STATEMENT

None.

## INFORMED CONSENT STATEMENT

I confirm that written patient consent has been signed and collected in accordance with the journal's patient consent policy.

## Data Availability

None.
